# Health Care System Overstretch and In-Hospital Mortality of Intubated Patients With COVID-19 in Greece From September 2020 to April 2022: Updated Retrospective Cohort Study

**DOI:** 10.2196/43341

**Published:** 2024-06-10

**Authors:** Theodore Lytras

**Affiliations:** 1 School of Medicine, European University Cyprus, Engomi, Cyprus

**Keywords:** COVID-19, pandemic, health care disparities, intensive care unit, right to health, quality of care, intubation, mortality, health disparity, health inequality, surveillance data, inpatient, mortality, COVID-19 patient, hospitalization, ICU, disparity, inequality, surveillance, health care system, Greece, region, Delta, Omicron, vaccination, vaccine, public health, patient load, deterioration, time

## Abstract

**Background:**

Our previous analysis showed how in-hospital mortality of intubated patients with COVID-19 in Greece is adversely affected by patient load and regional disparities.

**Objective:**

We aimed to update this analysis to include the large Delta and Omicron waves that affected Greece during 2021-2022, while also considering the effect of vaccination on in-hospital mortality.

**Methods:**

Anonymized surveillance data were analyzed from all patients with COVID-19 in Greece intubated between September 1, 2020, and April 4, 2022, and followed up until May 17, 2022. Time-split Poisson regression was used to estimate the hazard of dying as a function of fixed and time-varying covariates: the daily total count of intubated patients with COVID-19 in Greece, age, sex, COVID-19 vaccination status, region of the hospital (Attica, Thessaloniki, or rest of Greece), being in an intensive care unit, and an indicator for the period from September 1, 2021.

**Results:**

A total of 14,011 intubated patients with COVID-19 were analyzed, of whom 10,466 (74.7%) died. Mortality was significantly higher with a load of 400-499 intubated patients, with an adjusted hazard ratio (HR) of 1.22 (95% CI 1.09-1.38), rising progressively up to 1.48 (95% CI 1.31-1.69) for a load of ≥800 patients. Hospitalization away from the Attica region was also independently associated with increased mortality (Thessaloniki: HR 1.22, 95% CI 1.13-1.32; rest of Greece: HR 1.64, 95% CI 1.54-1.75), as was hospitalization after September 1, 2021 (HR 1.21, 95% CI 1.09-1.36). COVID-19 vaccination did not affect the mortality of these already severely ill patients, the majority of whom (11,944/14,011, 85.2%) were unvaccinated.

**Conclusions:**

Our results confirm that in-hospital mortality of severely ill patients with COVID-19 is adversely affected by high patient load and regional disparities, and point to a further significant deterioration after September 1, 2021, especially away from Attica and Thessaloniki. This highlights the need for urgent strengthening of health care services in Greece, ensuring equitable and high-quality care for all.

## Introduction

During the COVID-19 pandemic, an association between high patient load and in-hospital mortality has been identified in different settings [1,2]. We previously showed how the mortality of intubated patients with COVID-19 in Greece is affected by regional disparities and patient load, even without exceeding capacity [3]; that analysis did not consider COVID-19 vaccination and only covered the period until May 2021, thereby missing the large Delta and Omicron variant pandemic waves that followed, which were accompanied by a large number of deaths.

In this context, we aimed to update our analysis in order to (1) validate our previously published findings regarding the association between patient load, regional disparities, and mortality of intubated patients with COVID-19; (2) examine whether any changes in this association happened during the recent period, when Delta and Omicron were in circulation; and (3) examine whether vaccination reduces the mortality of already severely ill patients with COVID-19.

## Methods

### Overview

Our methods have been described before [3]; our study followed the retrospective cohort design. Briefly, we obtained anonymized patient data from the Greek National Public Health Organization (NPHO) for all cases intubated between September 1, 2020, and April 3, 2022, including vaccination status (number of doses received); dates of intubation, extubation, intensive care unit (ICU) admission, and discharge; and outcome at discharge (alive or dead); we followed these cases up to May 17, 2022.

Follow-up time between intubation and extubation or death was split finely into days, and Poisson regression was used to estimate the hazard of dying as a function of fixed and time-varying covariates [4]. Deaths occurring up to 5 days after extubation were classified as deaths at the end of follow-up. We included in the regression the following covariates: the daily total of intubated patients with COVID-19 as an indicator of health care system stress, age (as a natural cubic spline with 1 internal knot), sex, vaccination status (as a categorical variable), a linear time trend, ICU hospitalization (vs non-ICU), hospital region (the metropolitan regions of Attica and Thessaloniki vs the rest of Greece), an indicator for the period from September 1, 2021, and an interaction between period and hospital region. Model-based effect estimates were used to calculate population-attributable fractions [5] for the different covariates included.

### Ethical Considerations

No ethical approval was necessary as we used only anonymous surveillance data from which no patient could be identified. These data were provided to the author by the NPHO for epidemiological analysis purposes in the context of the COVID-19 pandemic response.

## Results

**Figure 1.**
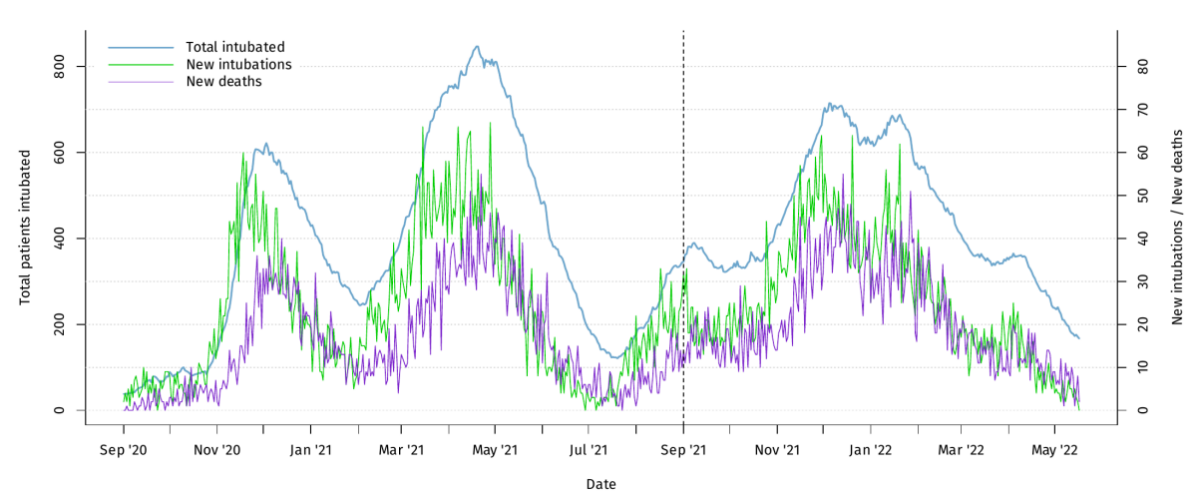
Distribution over time of total intubated patients with COVID-19, new intubations, and deaths among the study population in Greece from September 1, 2020, to May 17, 2022.

The distribution over time of new intubations, deaths among the study population, and total intubated patients with COVID-19 is illustrated in Figure 1. From August 2021, intubations and deaths gradually increased concurrently with the increased circulation of the Delta variant, especially so from November 2021. Then, after January 2022, circulation of the Omicron variant prolonged this epidemic wave further, with gradual de-escalation until the end of the study period.

A total of 14,011 intubated COVID-19 patients were analyzed, of whom 10,466 (74.7%) died and 11,944 (85.2%) were unvaccinated (Table 1). Most patients spent part or all of their hospital stay in an ICU (12,902/14,011 patients, representing 239,201/250,978 person-days in total). Among those not admitted in an ICU, nearly all died (1084/1109 patients, 97.7%), compared to 72.7% (9382/12,902 patients) among those admitted (*P*<.001).

**Table 1.** Characteristics of the study population: patients with laboratory-confirmed COVID-19 in Greece intubated between September 1, 2020, and April 3, 2022.

Characteristics		Total participants (N=14,011)	Surviving participants (n=3545)^a^	Deceased participants (n=10,466)^a^	*P* value^b^
Age (years), median (IQR)		68 (59-75)	60 (52-68)	70 (62-77)	<.001
**Sex, n (%)**					.03
	Female	5312 (100)	1290 (24)	4022 (76)	
	Male	8699 (100)	2255 (26)	6444 (74)	
**Vaccination status, n (%)**					<.001
	Unvaccinated	11,944 (100)	3157 (26)	8787 (74)	
	Vaccinated with 1 dose	574 (100)	127 (22)	447 (78)	
	Vaccinated with 2 doses	1054 (100)	175 (17)	879 (83)	
	Vaccinated with 3 doses	439 (100)	86 (20)	353 (80)	
**Hospitalization type, n (%)**					<.001
	In the ICU^c^	12,902 (100)	3520 (27)	9382 (73)	
	Outside the ICU	1109 (100)	25 (2)	1084 (98)	
**Time period, n (%)**					<.001
	After September 1, 2021	6163 (100)	1280 (21)	4883 (79)	
	Until August 31, 2021	7848 (100)	2265 (29)	5583 (71)	
**Hospital region, n (%)**					<.001
	Attica	5892 (100)	1817 (31)	4075 (69)	
	Thessaloniki	3065 (100)	760 (25)	2305 (75)	
	Rest of Greece	5054 (100)	968 (19)	4086 (81)	
**Total number of people intubated (at date of intubation), n (%)**					<.001
	0-199	767 (100)	277 (36)	490 (64)	
	200-299	821 (100)	284 (35)	537 (65)	
	300-399	2824 (100)	756 (27)	2068 (73)	
	400-499	1648 (100)	442 (27)	1206 (73)	
	500-599	2055 (100)	459 (22)	1596 (78)	
	600-699	3300 (100)	708 (21)	2592 (79)	
	700-799	1686 (100)	396 (23)	1290 (77)	
	≥800	910 (100)	223 (25)	687 (75)	

a The denominator for the percentages in these columns is the value in the Total column.

b Mann-Whitney test for continuous variables; Fisher test for categorical variables

c ICU: intensive care unit.

**Figure 2.**
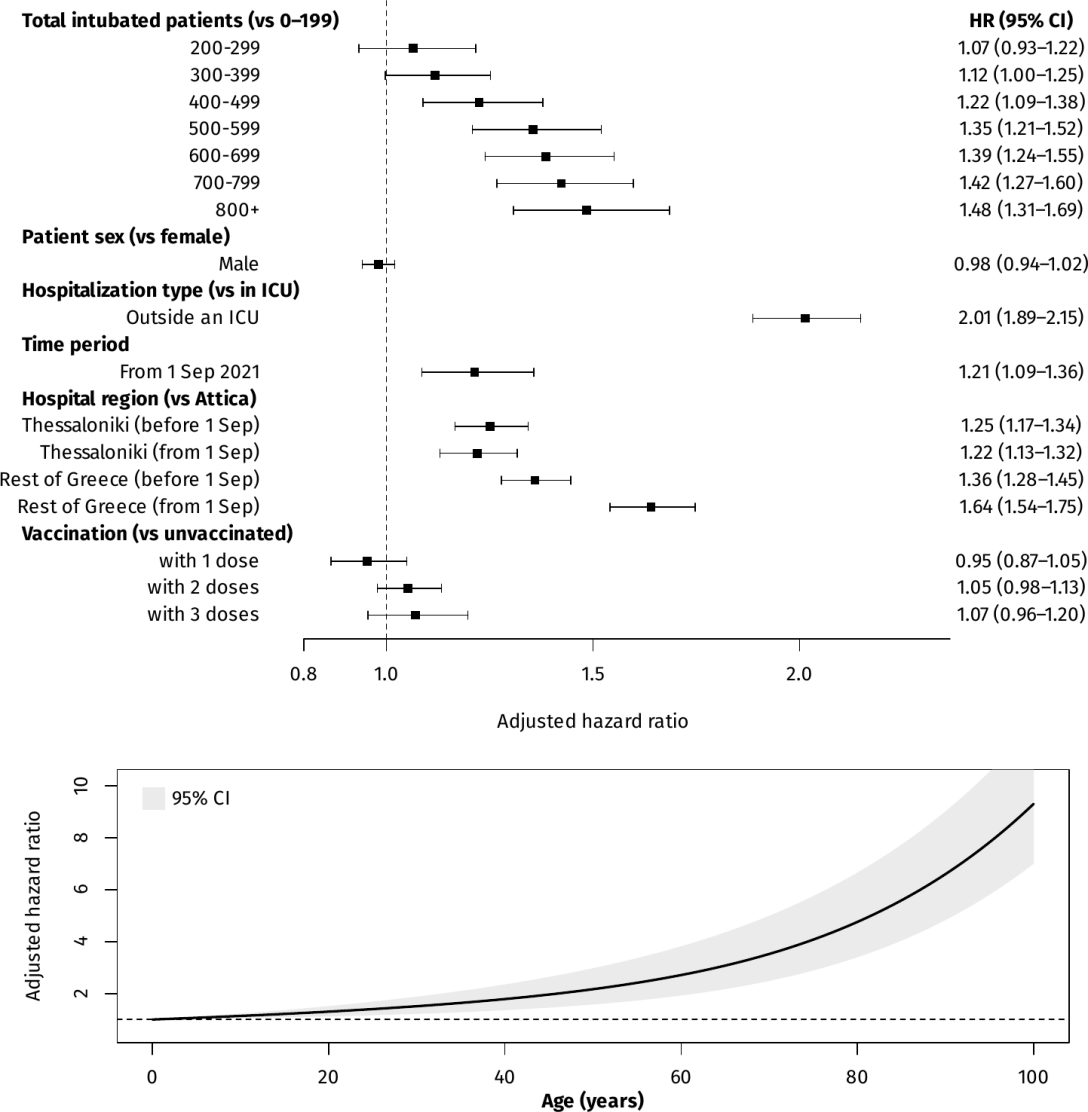
Multivariable (adjusted) associations between in-hospital mortality of intubated patients with COVID-19 and age, sex, hospitalization type, hospital region, and patient load in Greece from September 1, 2020, to May 17, 2022. HR: hazard ratio; ICU: intensive care unit.

All model results, expressed as adjusted hazard ratios (HRs) and 95% CIs, are shown in Figure 2. There was a significant association between mortality and a total number of intubated patients above 400, with its magnitude increasing progressively: from 1.22 (95% CI 1.09-1.38) for 400-499 patients, up to 1.48 (95% CI 1.31-1.69) for ≥800 patients. Interestingly, for a given patient load there was substantially increased mortality for the period after September 1, 2021, with an HR of 1.21 (95% CI 1.09-1.36). Being hospitalized outside the capital region of Attica was also associated with increased in-hospital mortality, especially for the rest of the country (besides Attica and Thessaloniki) and even more so after September 1, with an HR of 1.64 (95% CI 1.54-1.75). In addition, age was strongly associated with mortality, as was being intubated outside an ICU (HR 2.01, 95% CI 1.89-2.15). Vaccination did not show a statistically significant association with mortality, regardless of the number of doses received (Figure 2).

Given the above associations, of the 10,466 deaths reported, 2176 (95% CI 1297-2986) were attributable to the high load (≥200) of intubated patients with COVID-19, 564 (95% CI 499-634) to being outside an ICU, 1786 (95% CI 1572-2002) to being hospitalized away from Attica, and 1196 (95% CI 779-1586) to being hospitalized after September 1, 2021, and thus experiencing increased mortality. A combined total of 4677 deaths (95% CI 4021-5284) were attributable to all 4 factors collectively.

## Discussion

Our analysis confirms the earlier findings of our previously published study, indicating that in-hospital mortality of severely ill patients with COVID-19 is adversely affected by high patient load [3]; indeed, the associations between patient load and mortality were nearly identical to our previous analysis but now with significantly higher precision (narrower 95% CIs) as a result of a much larger sample (14,011 patients compared to 6282 previously). This shows that patient outcomes are affected not just when health care capacity is stressed to depletion, but also at lower levels, despite the availability of care not nominally being restricted. This represents a major preventable factor to limit avoidable deaths from COVID-19, and points toward more extensive investment in preparedness and resilience in health care. We also reconfirmed substantial regional disparities, with in-hospital mortality being lower in Thessaloniki and even lower in Attica compared to the rest of the country, highlighting the chronically uneven regional distribution of health care resources in Greece [6].

A major new finding is the 21% higher mortality observed in the period starting September 2021, which increased even further for patients hospitalized in the rest of the country (an additional +64%, compared to +36% for the previous period). This suggests that conditions in health care services over the past year may have further deteriorated, especially in rural areas (outside Attica and Thessaloniki). It must be noted that from September 2021 the government suspended those health care workers that remained unvaccinated against COVID-19 (although many of them eventually received vaccinations and returned to duty or were replaced with newly hired personnel). Our data cannot prove a causal association between this disciplinary action and the increased mortality, but the temporal coincidence is still worrisome and merits further exploration into the precise causes of this worsening health care system performance.

Our analysis reconfirms that being intubated outside an ICU is associated with twice higher mortality than inside an ICU, although again this must be interpreted with caution; patient selection (prioritizing those with a higher chance of survival for ICU admission) may account for a large part of this association. On the other hand, we found no statistically significant association between mortality and the number of COVID-19 vaccine doses received; this suggests that despite vaccination being enormously effective in preventing severe COVID-19 and death [7], if a patient has already been intubated as a result of severe COVID-19, it is quality of care and not vaccination that can prevent further deterioration and death.

In fact, the observed nonsignificant trend toward higher mortality among those having received 2 or 3 vaccine doses may be the result of some uncontrolled confounding due to comorbidities, as older people and those with comorbidities tend to be more frequently vaccinated. Indeed, the lack of information on comorbidities and baseline health status is the single and most significant limitation of our analysis; nevertheless, these factors are unlikely to be differentially distributed over time and with different patient loads. Both factors are also eclipsed by age, which is the major determinant of the risk of death and which we carefully adjusted for [8,9]. Therefore, residual confounding by baseline health is unlikely to have affected these results to any substantial extent.

As a result, the observed associations most likely reflect real and avoidable differences in the quality of care for patients with COVID-19 due to increased patient load, regional disparities, and ICU availability, as well as a true deterioration after September 1, 2021. The findings highlight the need for urgent strengthening of health care services in Greece in order to improve their performance and ensure equitable access to high-quality care for all [10].

## Supplementary material

10.2196/43341 Multimedia Appendix 1 Study data set (CC BY-NC).

## Abbreviations

HR: hazard ratio
ICU: intensive care unit
NPHO: National Public Health Organization
